# Diversity and Distribution Patterns of Invasive Alien Plant Species Along Dispersal Corridors in Parsa National Park, Central Nepal

**DOI:** 10.1002/ece3.73046

**Published:** 2026-02-01

**Authors:** Shreehari Bhattarai, Balram Bhatta, Jeetendra Gautam, Bharat Babu Shrestha

**Affiliations:** ^1^ Faculty of Forestry Agriculture and Forestry University Hetauda Nepal; ^2^ Central Department of Botany Tribhuvan University Kathmandu Nepal

**Keywords:** ecotourism, IAPS, protected area, tropical forest

## Abstract

Protected areas are vital for preserving native biodiversity, yet invasive alien plant species (IAPS) pose significant threats to their conservation. This study investigates how IAPS diversity varies across their dispersal corridors, such as road verges, riversides, seasonal springs, fire lines, and trekking trails, and examines the relationship between tree canopy openness and IAPS richness. We sampled 156 plots (2 m × 5 m) along these corridors in Parsa National Park (PNP), ensuring a minimum distance of 100 m between plots. Out of 10 IAPS recorded during the study, road verges and riversides were invaded by 70% of the recorded species, while the walking trail had the lowest number of IAPS (30%). The most prevalent IAPS was *Chromolaena odorata* , followed by *Mikania micrantha* and *Ageratum conyzoides* . One‐way ANOVA indicated significant differences in IAPS richness among dispersal corridors (*p* < 0.001). Furthermore, IAPS richness increased with increasing tree canopy openness (*R* = 0.8, *p* < 0.001). Given that road verges and riversides appeared to be the major dispersal corridors of IAPS in the context of PNP, it is imperative to prioritize these corridors for IAPS monitoring, early detection, eradication, and control. Such efforts can reduce the establishment probability of new IAPS and mitigate the impacts of the widespread IAPS on the native species and ecosystems.

## Graphical abstract

Protected areas face growing pressure from invasive alien plant species (IAPS), whose distribution differs across dispersal corridors. This study reveals that road verges and riversides were the most heavily invaded areas (70% of recorded species), with *Chromolaena odorata* being the most dominant IAPS. Additionally, richness increased significantly with increasing canopy openness. These results underscore the importance of prioritizing road verges and riversides for focused IAPS monitoring, early detection, and management to safeguard native biodiversity.

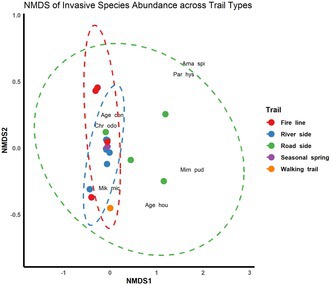

## 1 Introduction

Protected areas, such as national parks, are designed to protect native biodiversity and ecosystems while also providing social and economic benefits to local communities and visitors. These areas have multiple objectives, including biodiversity conservation, the protection of cultural heritages, scientific research, recreation, and ecotourism (Esfehani and Albrecht 2018). Many protected areas also offer important cultural ecosystem services, such as educational opportunities, spiritual enrichment, and recreational activities like hiking and wildlife watching. Protected areas are vital for maintaining natural landscapes and the ecosystem's integrity, playing a key role in safeguarding species and their habitats (Braun et al. 2016; Chape et al. 2005; Tittensor et al. 2014). However, many protected areas are under threat of multiple factors, including the spread of Invasive Alien Plant Species (IAPS) (Foxcroft et al. 2011). Plant species that come from one geographic area but are introduced to other regions, where they establish, spread, and harm biodiversity, ecosystem functions, and human well‐being, are called IAPS (CBD 2002). These species often have traits that increase their invasiveness. These traits include strong allelopathic effects, fast vegetative growth, high seed production, long‐lasting seed banks, early sexual reproduction, notable phenotypic plasticity, and the ability to thrive in a wide range of environmental conditions (Rai and Tripathi 1982). The IAPS can have detrimental impacts, affecting native biodiversity, ecosystem health, people's livelihoods, agriculture, and aquaculture, while also causing economic damage (Aerts et al. 2017; Bellard et al. 2016; Hattab et al. 2017; Rai and Singh 2020; Ricotta et al. 2010). In some protected areas, ecotourism, which is often considered as a sustainable means of financing conservation efforts, can unintentionally facilitate IAPS introduction and spread by introducing potentially invasive species into otherwise pristine environments (Tu and Robison 2013). The encroachment of IAPS into protected areas is an emergent global concern and is increasingly recognized by conservation professionals as a serious and growing threat (Foxcroft et al. 2017; Padmanaba et al. 2017).

Their rapid spread, high reproductive capacity, adaptability to new environments, and competitive dominance over native species have led to habitat degradation and ecosystem disruption. In Nepal, the spread of invasive species such as *Chromolaena odorata* , *Lantana camara* , *Mesosphaerum suaveolens*, *Mikania micrantha* , and *Parthenium hysterophorus* is becoming an increasing concern, especially within lowland protected areas (Murphy et al. 2013; Sapkota 2007; Shrestha et al. 2025). To date, 18 naturalized plant species belonging to 49 families and 129 genera have been recorded in Nepal (Shrestha 2019; Shrestha and Shrestha 2021), many of which pose potential ecological threats to the protected areas of Nepal (Bhatta et al. 2020, 2024; Shrestha et al. 2025). In particular, the Parsa National Park has already been invaded by more than two dozen of the IAPS, which have reduced regeneration of native trees, altered species composition of plant communities, and modified soil chemistry (Chaudhary et al. 2020). A recent study has also revealed that the IAPS in the PNP are affecting habitat utilization by wild ungulates (Rawal et al. 2025).

Within the landscape, including protected areas, dispersal of IAPS propagules often occurs along human‐made linear infrastructure such as roads and trails, and natural corridors such as springs and rivers (Brundu et al. 2025; Shrestha et al. 2018), though the protected area boundary may buffer, to some extent, the spread of IAPS to the core areas (Bhatta et al. 2024). Micro‐habitats along these linear structures also provide a conducive environment for the establishment of new IAPS, from where they subsequently spread into the landscape (Adhikari et al. 2024). In particular, roads serve as ideal environments and pathways for the spread of IAPS, playing a key role in their establishment and distribution (Le et al. 2018; Meunier and Lavoie 2012). While proximity to roads, rivers, and settlements has been identified as a major driver of IAPS occurrence, trail‐related disturbances have also been shown to facilitate invasion, often leading to the displacement of native flora (Bhatta et al. 2020; Chaudhary et al. 2020). However, a significant gap remains in mapping the spatial distribution of IAPS and understanding their spread along roads and trails within protected areas (Le et al. 2018; Shrestha et al. 2017), which limits the capacity of park authorities to develop and implement effective, evidence‐based management strategies (Shrestha et al. 2019). Despite several research and conservation works focusing on protected areas of Nepal, there is limited information on how linear infrastructure, particularly roads and trails, influences the distribution of IAPS in protected areas of Nepal. In this context, we aim to (1) examine how diversity of IAPS varies across human‐made and natural dispersal corridor types in Parsa National Park, and (2) understand how tree canopy openness affects IAPS diversity across potential dispersal corridors. These data can help implement site‐ and species‐specific approaches for the management of IAPS in Parsa National Park and other similar protected areas.

## 2 Materials and Methods

### 2.1 Study Area

**FIGURE 1:**
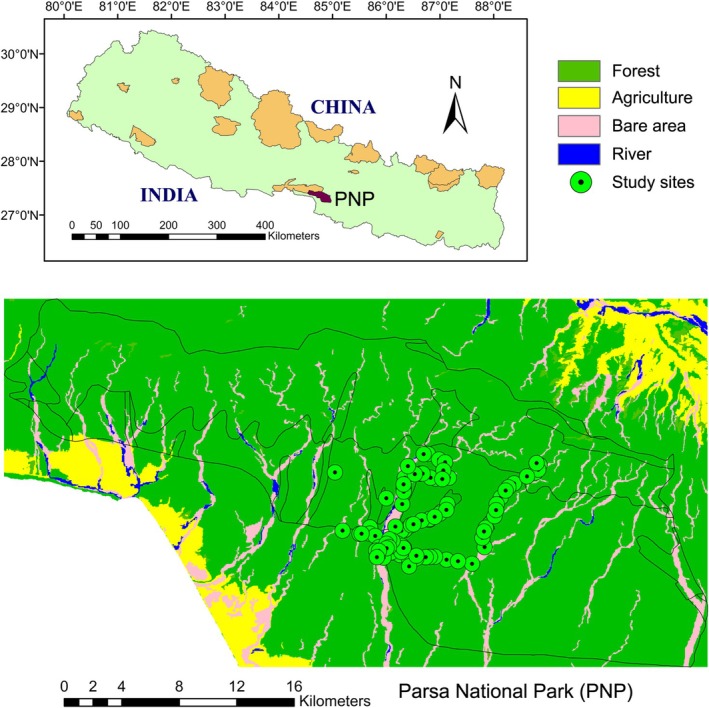
Map of Parsa National Park showing study sites.

Parsa National Park (PNP) is located in the south‐central lowland Tarai region of Nepal, covering the area between 27°15′ to 27°33′N latitude and 84°41′ to 84°58′ E longitude and ranging in elevation from 100 to 900 m (Figure 1). Initially established as a wildlife reserve in 1984 to protect the habitat of wild Asian elephants, it was designated a national park in 2017, covering an area of 627.39 km^2^. To the west of PNP, there is another protected area, the Chitwan National Park, which is also a World Heritage site. The dominant tree species in the park is Sal ( *Shorea robusta* ). The soil in the area is primarily gravelly and highly porous, characterized by a low water table, with many streams disappearing into the permeable sediments. Despite this, the park supports a rich biodiversity, including 298 species of vascular plants, 37 mammals, 503 birds, 8 species of herpetofauna, and 8 species of fish (Bhuju et al. 2007). The Parsa‐Chitwan Complex spans the Himalayan foothills and includes floodplains, Dun valleys, the Bhabar tract, and the Siwalik and Chure hills.

*Shorea robusta* forest is the principal vegetation type and is widely distributed, although species composition varies with slope, elevation, soil characteristics, and local microclimatic conditions. Mixed deciduous riverine forests are prominent along river corridors and are characterized by species such as *Dalbergia sissoo* , *Senegalia catechu*, *Bombax ceiba* , *Grewia disperma*, and *Holarrhena pubescens* , accompanied by shrub species like *Zyzyphus mauritiana*, *Caesalpinia acucullata*, and *Murraya koenigii* . In addition, mixed deciduous hardwood forests occur across upland areas comprising *Shorea robusta* , *Adina cordifolia*, *Terminalia alata*, *Dillenia pentagyna*, *Lagerstroemia parviflora*, *Mallotus philippensis* , and *Asparagus racemosus*, along with climbers such as *Smilax ovalifolia* and juvenile *Phoenix* species. *Chromolaena odorata* , *Mikania micrantha* , *Lantana camara* , *Senna tora* , *Ageratum conyzoides* , and *Parthenium hysterophorus* are the problematic IAPS in PNP (Bhuju et al. 2013; Chaudhary et al. 2020).

### 2.2 Data Collection

In forest landscapes, trails are a major type of disturbance. Their establishment creates canopy gaps and changes microclimatic conditions by increasing light, temperature, and wind exposure compared to undisturbed forest interiors (Godefroid and Koedam 2004). Recreational activities on trails, such as hiking, mountain biking, and horse riding, further increase disturbance through trampling, soil compaction, erosion, and direct damage to vegetation along trail surfaces and edges (Barros et al. 2013). These disturbances cause noticeable changes in vegetation, including less plant cover and height, changes in species composition, and more disturbance‐tolerant or invasive species. A reconnaissance survey was carried out with PNP staff to identify suitable trails in the park's core area. Trail types were classified into five categories based on their physical features and sources of disturbance: permanent riversides with year‐round flowing water, seasonal springs with intermittent flow, fire lines that are cleared strips for fire management, gravel roads as human‐made routes with gravel surfaces, and walking trails as narrow, low‐use footpaths. Transect lines were set up along the Bhata‐Laukidaha, Bhata‐Amlekhgunj fire line, Rambhouri‐Ghodemasan, Bhata‐Rambhouri, Bhata Range Post‐Dharmasala, Bhata‐Devakidaha, Bhata‐Jamini River, Bhata‐Chabi River, and nearby grassland areas. These locations also matched existing park security posts. Overall, 156 sampling plots (2 × 5 m) were laid out at intervals of over 100 m along major dispersal corridors. This included permanent riverbanks (*N* = 34), seasonal springs (*N* = 32), fire lines (*N* = 30), graveled roads (*N* = 27), and walking trails (*N* = 33). Tree canopy cover within each plot was measured using a spherical densitometer (Lemmon 1956).

### 2.3 Data Analysis

The frequency and abundance of each species were calculated using the methods described in (Dallmeier 1992; Shukla and Chandel 1994), whereas the IAPS diversity index was calculated using the Shannon‐Wiener biodiversity index.

$$\text{Frequency}\;\left(\%\right)=\frac{\text{Total number of quadrats in which the species occur}}{\text{Total number of quadrats studied}}\times 100$$

$$\text{Abundance}/\mathrm{ha}=\frac{\text{Total number of individuals of}\;a\;\text{species in}\;\mathrm{all}\;\text{the quadrats}}{\text{Total area of quadrats in which the species occurred}}\times 10000$$

$$\text{Shannon}-\text{Wiener biodiversity index}\;\left(H^{\prime }\right)=-\mathrm{\Sigma pi}\times \text{lnpi}$$

where H′ is the diversity index, pi is the proportion of *i*th species individuals to total species individuals, and ln is the natural logarithm.

The one‐way ANOVA was used to compare the IAPS richness among different trail types. The influence of canopy openness (%) on IAPS richness was assessed using simple linear regression analysis at a 5% level of significance. To assess patterns in IAPS community composition across different trail types, a Non‐metric Multidimensional Scaling (NMDS) analysis was performed (Bray and Curtis 1957; Kuswantoro et al. 2020). The Bray–Curtis dissimilarity index was used to construct the distance matrix. NMDS ordination was then carried out using a two‐dimensional solution to provide a simplified yet accurate representation of multivariate relationships among sites. Ordination stress values were examined to evaluate the adequacy of the two‐dimensional configuration, with stress values close to or less than 0.2 considered to indicate an excellent representation of the underlying data structure (Clarke 1993; Dhakal et al. 2024). Site scores were overlaid with trail type groupings to visualize clustering patterns in species composition among Fire line, Roadside, Riverside, Walking trail, and Seasonal spring categories. The analysis was performed in R (version 2025.05.1) using the *vegan* package.

## 3 Results

### 3.1 IAPS Richness Along Different Trails

A total of 10 IAPS belonging to five families were found along the trails in PNP (Table 1). Asteraceae was the dominant family, with five species, followed by Fabaceae (3), while Rubiaceae and Amaranthaceae each had one species. Most species came from Central and South America, showing a strong influence from the Neotropics.

**TABLE 1:** Checklist of IAPS recorded along trails in PNP.

S. no.	Species	Local name	Common name	Family	Native range
1	*Chromolaena odorata* (L.) R. M. King & H. Rob.	Seto Banmara	Siam weed	Asteraceae	Central and tropical South America
2	*Spermacoce alata* Aubl.	Aalupate	Winged false button	Rubiaceae	West Indies and tropical America
3	*Senna tora* (L.) Roxb.	Sano Tapre	Sickle Senna/pod	Fabaceae	Central America
4	*Mikania micrantha* Kunth	Lahare Banmara	Mile‐a‐minute, American rope	Asteraceae	Tropical and subtropical regions of Central America, South America
5	*Ageratum conyzoides* L.	Seto Gandhe	Billygoat weed, White weed	Asteraceae	Central and South America
6	*Ageratum houstonianum* Mill.	Nilo Gandhe	Floss flower, Blue mink, Blue billy‐goat weed	Asteraceae	Central and Southern Mexico
7	*Mimosa pudica* L.	Lajjawati	Touch‐me‐not plant	Fabaceae	Parts of Northern and Southern America, including Mexico, Brazil, Caribbean; Tropical region
8	*Parthenium hysterophorus* L.	Paati Jhar, Kanike Jhar, Gajare Jhar	Congress Grass, Carrot Grass, Parthenium Weed, Whitetop	Asteraceae	Tropical America
9	*Amaranthus spinosus* L.	Lunde Kanda	Spiny Pig‐weed	Amaranthaceae	Tropical America
10	*Senna occidentalis* (L.) Link	Thulo Tapre	Coffee Senna, Negro Coffee	Fabaceae	Tropical America

**FIGURE 2:**
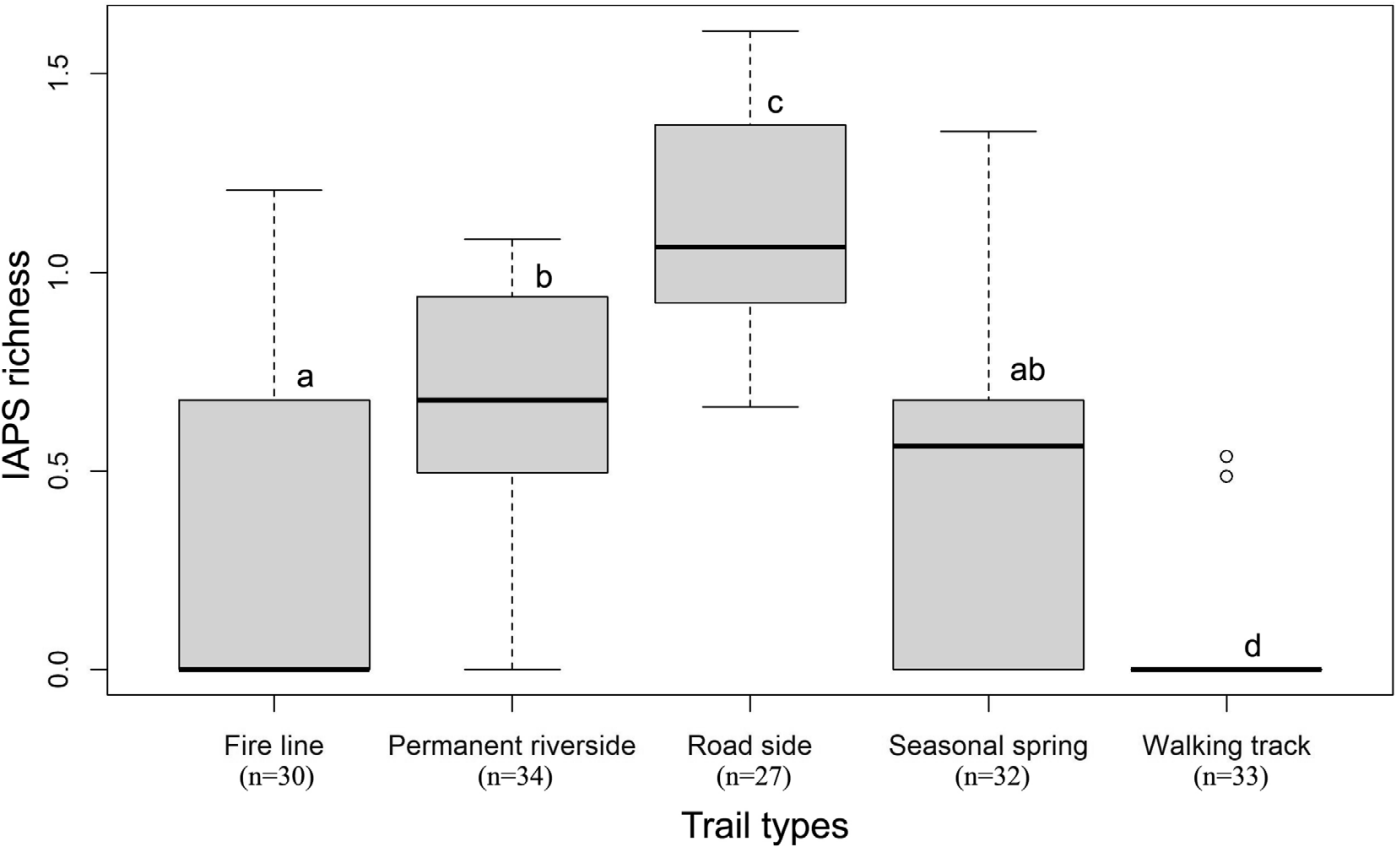
IAPS richness along different trail types.

The one‐way ANOVA revealed significant variation in IAPS richness among different trail types (*F* = 15.70, *p* < 0.001). Tukey's post hoc comparisons showed that roadside habitats supported significantly higher IAPS richness compared to permanent riversides, seasonal springs, and walking tracks, while the walking tracks harbored the lowest richness, being significantly different from all other trail types (Figure 2). Permanent riverside, seasonal spring, and fire line trails exhibited intermediate and statistically comparable levels of IAPS richness.

**FIGURE 3:**
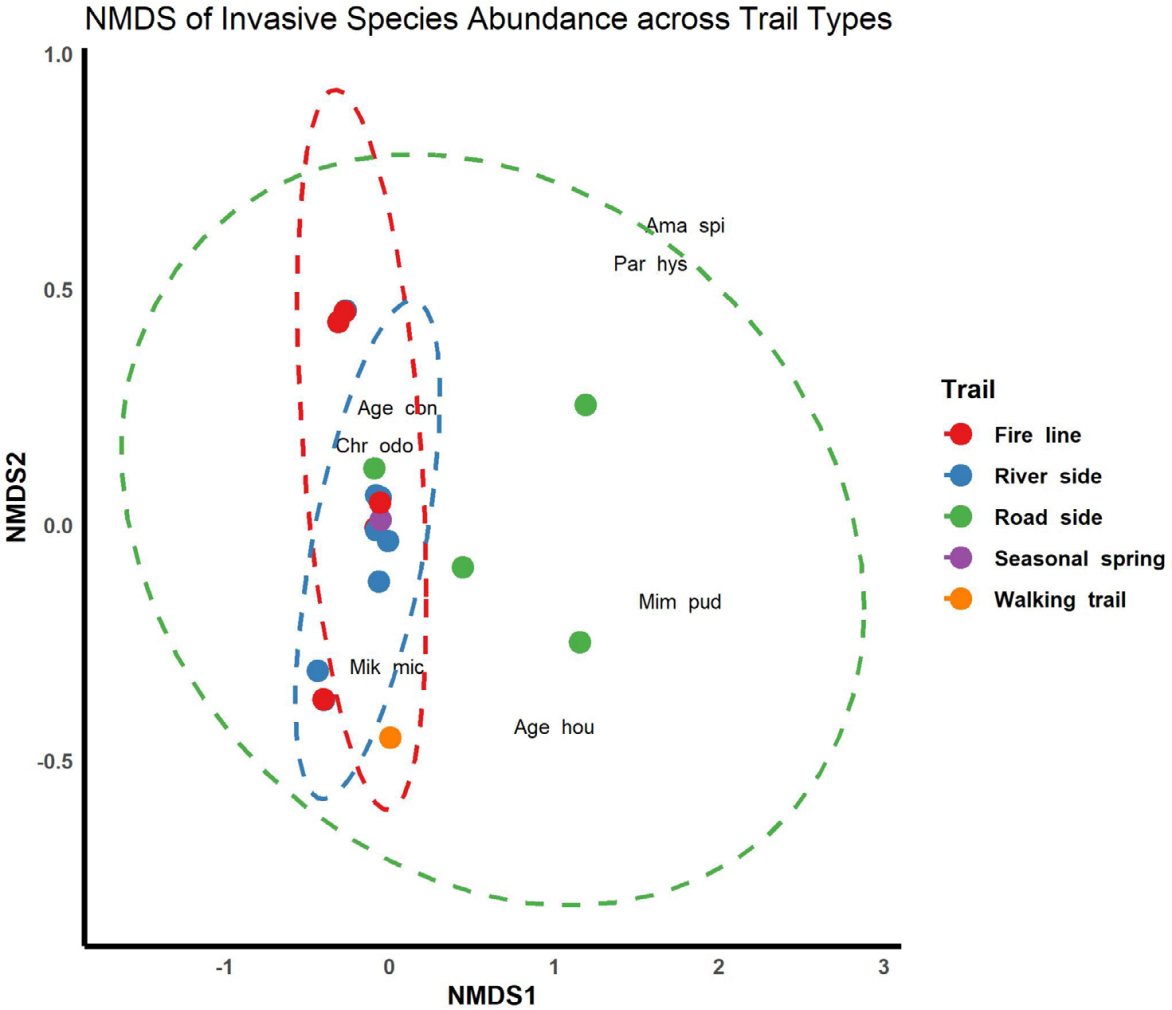
NMDS of invasive alien plant species abundance across trail types.

The Non‐metric Multidimensional Scaling (NMDS) analysis of invasive species abundance across different trail types revealed clear patterns in community composition (Figure 3). The NMDS ordination achieved a stress value of 0.059, indicating an excellent fit of the two‐dimensional representation to the original multidimensional data. Sites belonging to similar trail types tended to cluster together, suggesting comparable species composition within each trail category. For example, Fire line and Roadside sites clustered closely, driven primarily by high abundances of *Chromolaena odorata* , *Mikania micrantha* , and *Ageratum conyzoides* . River‐side sites formed a distinct cluster, reflecting differences in dominant species composition relative to other trail types. Walking trail and Seasonal spring sites were more dispersed, indicating greater variability in species abundance.

### 3.2 Frequency and Abundance of IAPS Along Different Trails

The frequency and abundance data demonstrated clear habitat‐wise dominance patterns (Table 2). *Chromolaena odorata* exhibited the highest overall frequency and consistently high abundance, occurring in all roadside plots (100%) and showing very high frequencies along permanent riversides (87.5%) and seasonal springs (80.56%). Its abundance was also high across habitats, reaching 14,380/ha along permanent riversides, followed by fire lines (14,330/ha) and roads (13,500/ha), confirming its broad ecological tolerance and strong invasive potential. *Ageratum conyzoides* ranked next in dominance, showing high frequency along roads (87.5%) and permanent riversides (54.17%), and moderate frequency along fire lines (34.38%). Despite lower frequency along walking tracks (8.7%), it attained the highest abundance there (22,000/ha), followed by fire lines (17,550/ha) and roads (17,290/ha), indicating strong local dominance in highly disturbed habitats. Similarly, *Mikania micrantha* also showed relatively high frequency along roads (100%) and permanent riversides (66.67%), with decreasing frequency toward seasonal springs (41.67%), fire lines (28.13%), and walking tracks (13.04%). Its abundance remained high across habitats, particularly along permanent riversides (12,690/ha) and walking tracks (10,330/ha), reflecting its adaptability to both moist and disturbed environments.

**TABLE 2:** Frequency and abundance of IAPS along different trails.

S. no.	Species	Permanent riverside		Seasonal spring		Fire line		Road		Walking track	
		Frequency (%)	Abundance/ha	Frequency (%)	Abundance/ha	Frequency (%)	Abundance/ha	Frequency (%)	Abundance/ha	Frequency (%)	Abundance/ha
1	*Chromolaena odorata* (L.) R. M. King & H. Rob.	87.5	14,380	80.56	10,070	56.25	14,330	100.00	13,500	8.7	9500
2	*Spermacoce alata* Aubl.	0	0	2.78	2000	0	0	0.00	0	0	0
3	*Senna tora* (L.) Roxb.	0	0	2.78	3000	0	0	0.00	0	0	0
4	*Mikania micrantha* Kunth	66.67	12,690	41.67	8930	28.13	9440	100.00	7130	13.04	10,330
5	*Ageratum conyzoides* L.	54.17	18,920	16.67	13,000	34.38	17,550	87.50	17,290	8.7	22,000
6	*Ageratum houstonianum* Mill.	0	0	0	0	0	0	25.00	17,000	0	0
7	*Mimosa pudica* L.	0	0	0	0	3.13	2000	25.00	3500	0	0
8	*Parthenium hysterophorus* L.	0	0	0	0	0	0	25.00	3500	0	0
9	*Amaranthus spinosus* L.	0	0	0	0	0	0	12.50	7000	0	0
10	*Senna occidentalis* (L.) Link	4.17	2000	25	5670	6.25	6000	0.00	0	0	0

On the contrary, species with lower frequency and abundance showed strong habitat specificity. *Ageratum houstonianum* , *Mimosa pudica* , and *Parthenium hysterophorus* occurred only along roads with a frequency of 25%, showing abundances of 3500–17,000/ha, while *Amaranthus spinosus* occurred in 12.5% of road plots with an abundance of 7000/ha. *Spermacoce alata* and *Senna tora* were the least represented species, each restricted to seasonal springs with very low frequency (2.78%) and low abundance (2000–3000/ha).

### 3.3 Effects of the Canopy on the IAPS Richness

**FIGURE 4:**
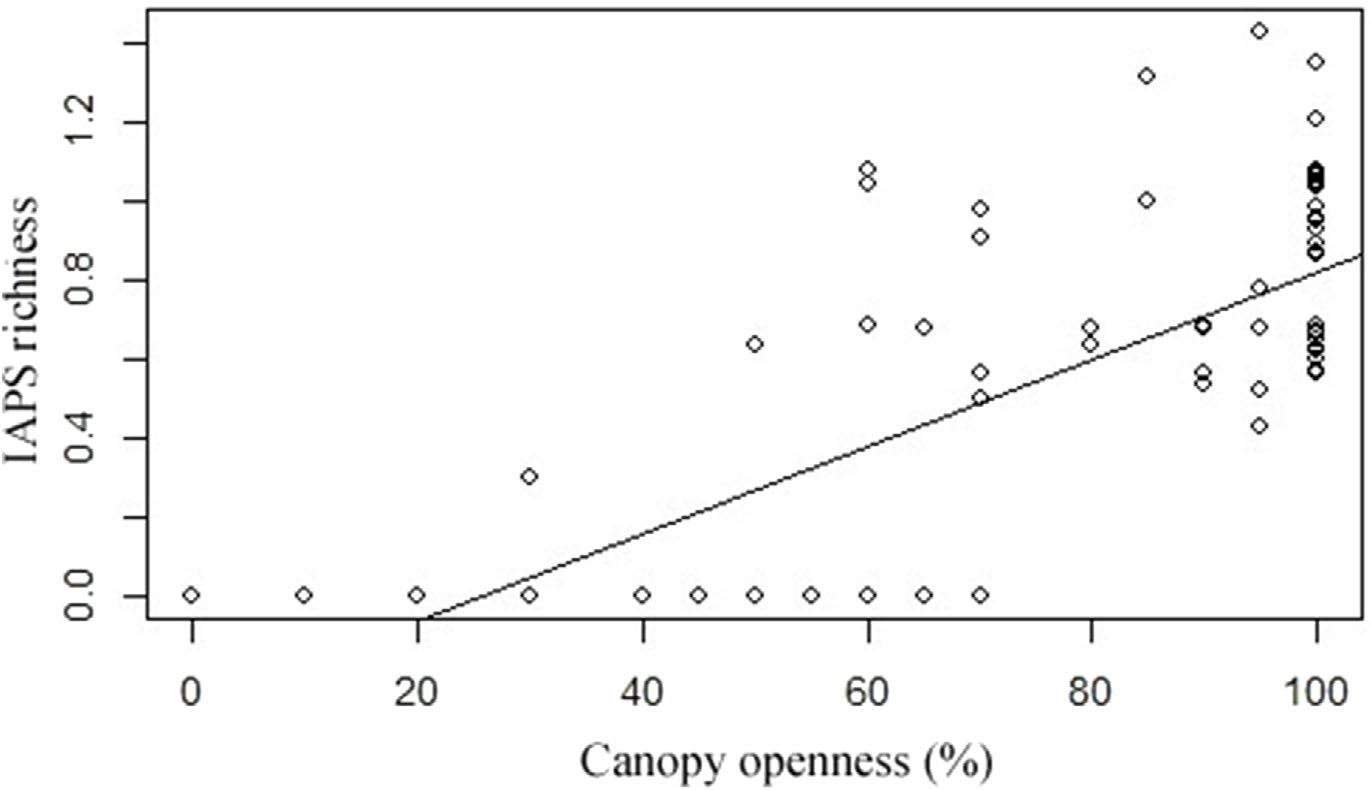
Influence of canopy openness along sampling plots (*n* = 156) on IAPS richness.

The influence of canopy openness (%) on the IAPS richness revealed a strong and statistically significant (*R* = 0.8, *p* = 0.000) role. At 100% canopy openness, there was a notable increase in IAPS diversity (Figure 4), indicating that fully open canopies support higher levels of invasive species diversity. Conversely, as canopy openness decreased, IAPS diversity also declined significantly, with many areas showing no invasive species under very dense canopy conditions. Although severalIAPS exist in Parsa National Park, their ecological impacts differ widely among species. *Chromolaena odorata* is one of the most troublesome IAPS in Nepal and is ranked among the world's 100 worst invasive species. Its high ability to adapt allows it to invade forests, shrublands, and grasslands. This invasion significantly reduces native species richness, alters community composition, and hinders the regeneration of key native species like *Shorea robusta* (Thapa et al. 2016; Bhatta et al. 2020; Chaudhary et al. 2020). *Mikania micrantha* , another widely recognized invasive species, threatens wildlife habitats, especially in protected areas (Bhatta et al. 2021). *Ageratum conyzoides* and *Ageratum houstonianum* also present a serious risk due to their toxic effects (Pertin et al. 2018; Sapkota 2007; Shrestha et al. 2019).

## 4 Discussion

### 4.1 Diversity and Distribution Pattern of IAPS Along Trail Types

The findings reveal a distinct pattern in the distribution of IAPS, with roadsides and permanent riversides identified as hotspots for both IAPS richness and abundance. The high proportion of alien species found along roads (70%) and permanent riversides (60%) highlights these environments as significant pathways for the spread of invasive species. Roads and rivers are typically associated with disturbances that promote the establishment of invasive species, including habitat fragmentation, altered microclimatic conditions, and increased human activity, which often serve as corridors for dispersal and provide favorable habitats for exotic species (Parendes and Jones 2000; Silva et al. 2017). The presence of roads and rivers can contribute to the invasion of protected areas in two key ways: by facilitating the migration of alien plants through external human activities, such as transportation by people and vehicles (Niggemann et al. 2009; Von der Lippe and Kowarik 2007), and by creating novel environments conducive to the establishment of alien species (Greenberg et al. 1997).

These results suggest that disturbance intensity and type strongly influence invasion patterns, with roadsides acting as major invasion hotspots, likely due to continuous anthropogenic disturbance and propagule pressure, whereas walking tracks are less susceptible to invasion (Laurance et al. 2009). Similar trends were observed along roads and permanent riversides, where trail users, particularly vehicles and runners, act as key vectors facilitating the spread of IAPS (Ngugi et al. 2014). Moderate levels of IAPS diversity recorded along seasonal springs and fire lines likely reflect regular movement of humans, livestock, and vehicles, conditions known to promote invasions under intermediate disturbance regimes (Doherty et al. 2021; Son et al. 2023). In contrast, the low IAPS diversity along walking trails can be attributed to limited connectivity and minimal disturbance (Doherty et al. 2021).

The widespread presence of *Chromolaena odorata* across all trails highlights its adaptability to highly disturbed environments, where it can outcompete native vegetation. Similarly, *Mikania micrantha* also demonstrates a strong presence, showcasing its ability to thrive in wet and disturbed conditions. The prevalence of these species in disturbed areas like roadsides and riversides indicates that such habitats offer ideal conditions for their rapid invasion and colonization. Their dominance poses significant ecological challenges, including the alteration of native plant communities, reduction in biodiversity, and interference with the regeneration of native species (Banerjee and Dewanji 2017). In contrast, walking tracks, which have only 30% IAPS and the lowest diversity index (0.053), reflect lower disturbance levels. These tracks, being narrower and less frequently disturbed compared to roads, provide fewer opportunities for the introduction and colonization of invasive species. This suggests that less‐disturbed habitats, such as walking tracks, maintain higher resistance to invasion, thereby preserving native species and sustaining ecological balance. In contrast, species like *Spermacoce alata* and *Senna tora* have minimal occurrences, mostly confined to seasonal springs with a low frequency of 2.78%. Other species, such as *Ageratum houstonianum* , *Mimosa pudica* , and *Parthenium hysterophorus* are limited to specific areas, particularly along roads with moderate to low frequencies. This pattern reveals a concentration of invasive species in highly disturbed areas like roads and riversides, while other trails, like walking tracks, exhibit much lower invasion rates. *Ageratum conyzoides* stands out with the highest abundance, particularly along walking tracks and fire lines, suggesting that species such as *Ageratum conyzoides* and *Chromolaena odorata* dominate the landscape. In contrast, other species exhibit more restricted distributions based on trail types.

Species labels on the NMDS plot highlight the key species contributing to community differences, whereas species absent from all sites were automatically excluded from the analysis. Overall, the NMDS results indicate that trail type has a strong influence on the composition of invasive plant communities, with certain species serving as indicators of specific disturbance regimes. These patterns are closely linked to dispersal pathways, as different trail types function as conduits for propagule movement. Roads and walking tracks, for example, facilitate seed dispersal through vehicular traffic, human movement, and maintenance activities, promoting the establishment of disturbance‐tolerant invasive species. Similarly, fire lines and riversides act as linear corridors that enhance species spread via wind, water flow, and repeated disturbance. The ellipses around each trail type further emphasize the clustering patterns, showing that sites within the same trail type share similar species assemblages due to common dispersal mechanisms and disturbance histories. Together, these results suggest that trail‐specific dispersal pathways play a key role in shaping invasive plant community composition across the landscape.

### 4.2 Relationship Between Canopy Openness and IAPS Richness

The strong positive correlation (*R* = 0.8, *p* = 0.000) between canopy openness and the diversity of IAPS highlights the crucial role of light availability in fostering the spread of these species. At 100% canopy openness, where light is plentiful, IAPS diversity reaches its peak, indicating that fully open environments are particularly vulnerable to invasion. Invasive species, which are often fast‐growing and require ample light, can establish and proliferate rapidly in open‐canopy areas where competition from native plants is diminished, and environmental conditions such as light, temperature, and moisture are more conducive to their growth (Le et al. 2018). Forest canopy cover is the overriding biotic covariate suppressing the diversity and distribution of IAPS (Sharma et al. 2022), and an increase in canopy cover and closure of forest gaps through proper management of degraded forests can prevent plant invasions and suppress the growth of previously established IAPS (Khaniya and Shrestha 2020; Adhikari et al. 2024). These results suggest that greater canopy openness is linked to higher IAPS diversity, while reduced canopy openness limits the presence and spread of invasive species, likely due to decreased light availability or other ecological constraints. Disturbances that increase canopy openness, such as logging, natural disasters, or land‐use changes, heighten the risk of IAPS invasion. Thus, maintaining or restoring forest ecosystems in degraded areas can be an effective strategy to curb the spread of invasive species and protect native biodiversity.

### 4.3 Implications for Conservation

Our findings show that the distribution and diversity of IAPS were mostly concentrated along roads, highlighting their role as major pathways for spreading within PNP. Vehicle movement, trail runners, and visitor traffic on these routes, along with open canopy areas and repeated disturbances, help with seed dispersal, establishment, and quick growth of IAPS. Furthermore, sporting and recreational activities in protected areas increase the risk of introducing alien plants, stressing the need for active management. Tourists may unintentionally transport seeds from one site to another. From an ecotourism viewpoint, more tourist access to protected areas makes them more vulnerable to alien species invasions (Charles and Dukes 2007; Pickering et al. 2007). The chance of invasive species spreading through tourists is particularly high and may also aid in spreading cross‐regional pathogens (Septiadi et al. 2018; Zuhri and Mutaqien 2013).

Given that roads and riversides function as effective invasion corridors, these areas should be prioritized for regular monitoring, early detection, and rapid eradication of IAPS. Integrating IAPS management into the park's management plan is therefore crucial to prevent further introductions and to safeguard native biodiversity. Management strategies should focus on biosecurity measures, habitat restoration, and awareness programs implemented in collaboration with ecotourism managers and park users. The results also have important implications for infrastructure planning within protected areas. Limiting new road construction is essential to reduce future invasion risk, while managing trail width and disturbance intensity can further minimize IAPS spread. Notably, narrow walking trails with lower user pressure supported fewer invasive species, suggesting that low‐impact trail design and controlled human movement can be effective tools in reducing invasion pressure. Overall, careful and targeted management of dispersal pathways is essential to mitigate the growing threat of IAPS in PNP.

## 5 Conclusions

The study underscores the critical role of disturbance regimes and canopy structure in shaping the diversity and distribution of invasive alien plant species (IAPS) within Parsa National Park. Roadsides and riversides emerge as primary invasion hotspots due to their openness and frequent disturbances, while intact forest canopies and minimally disturbed walking tracks exhibit higher resistance to invasion. The strong positive relationship between canopy openness and IAPS richness further highlights the vulnerability of degraded or open habitats to invasion. These findings emphasize the urgent need for integrated management approaches, including the restoration of forest canopy, targeted monitoring along dispersal corridors, and the implementation of strict biosecurity and awareness programs. Such efforts are essential to mitigate the spread of IAPS, safeguard native biodiversity, and maintain the ecological integrity of protected areas like PNP.

## Author Contributions

**Shreehari Bhattarai:** conceptualization (lead), data curation (lead), formal analysis (lead), methodology (equal), writing – original draft (lead), writing – review and editing (equal). **Balram Bhatta:** conceptualization (equal), investigation (equal), methodology (equal), supervision (lead), writing – review and editing (equal). **Jeetendra Gautam:** conceptualization (supporting), investigation (equal), methodology (supporting), writing – review and editing (equal). **Bharat Babu Shrestha:** conceptualization (equal), investigation (supporting), methodology (equal), supervision (lead), validation (lead), writing – review and editing (lead).

## Conflicts of Interest

The authors declare no conflicts of interest.

## Data Availability

The necessary data is included in the main text.
